# A new imperialist competitive algorithm with spiral rising mechanism for solving path optimization problems

**DOI:** 10.7717/peerj-cs.1075

**Published:** 2022-09-07

**Authors:** Xia Li, Junhan Chen, Lingfang Sun, Jing Li

**Affiliations:** School of Automation Engineering, Northeast Electric Power University, Jilin, China

**Keywords:** Intelligent optimization algorithm, Imperialist competitive algorithm, Path planning problem, Global optimization ability

## Abstract

Intelligent optimization algorithms have now become important means for solving global optimization problems. The imperialist competitive algorithm (ICA) is a nature-inspired meta-heuristic algorithm that imitates social behavior. ICA has been widely used in optimization problems, however, ICA tends to fall into a local optimal solution because of its fast convergence speed, which may lead to premature convergence when solving optimization problems. To solve these problems, a new improved ICA algorithm is proposed. Based on the original ICA algorithm, the theory of spiral rising is introduced to enlarge the search space and enhance the global search ability of the algorithm based on ensuring the necessary speed of convergence. In this paper, the improved optimization algorithm is applied to 19 classical benchmark functions, and the improved ICA is applied to the robot path optimization problems to solve the optimal path. The improved ICA algorithm improves the optimization ability and algorithm stability.

## Introduction

It is difficult to find global optimal values by using exact algorithms when solving the global optimization problems: the time-complexity is too high or it is impossible to find the global optimal solution. In recent years, approximation algorithms have begun to play their part in solving such problems, but where traditional approximation algorithms fail to meet research needs, meta-heuristics stand out because of their good optimization ability. These meta-heuristics have reasonably dealt with such inapproximable optimization problems, so more people have been involved in related research since their first development (Salmeron et al., 2019; Zhang & Wang, 2016).

In Fausto et al. (2020), meta-heuristics are classified into four categories according to the different basic concepts invoked thus: evolution-based, swarm-based, physics-based, and human behavior-based algorithms. In each of the different nature-inspired meta-heuristic algorithms the purpose is to obtain a search accuracy of the highest order, the fastest search speed, and the most applicable range of intelligent optimization algorithms. After these intelligent optimization algorithms are proposed, they are often applied to engineering optimization problems either alone or in combination with other algorithms (Hu et al., 2021; Yu et al., 2022).

The imperialist competitive algorithm (ICA) proposed by Atashpaz-Gargari & Lucas (2007) is a meta-heuristic algorithm imitating human social behavior. It involves simulating the colonial aggression of imperialist countries and the competitive mechanism of the law of the jungle to seek a local optimal value until finding the overall optimal value. It has achieved quite good optimization results in dealing with practical problems and compared with the previous proposed genetic algorithms (GA) (Holland, 1992) and particle swarm optimization (PSO) (Kennedy & Eberhart, 1995), it has improved the optimization accuracy and convergence speed to a certain extent; because of these, some practical engineering problems related to parameter optimization or function optimization use ICA to search for global optimal values.

ICA has been widely used in engineering practice due to its excellent optimization capability (Yan, Liu & Huang, 2022; Yu et al., 2022; Kashikolaei et al., 2020). Osmani, Mohasef & Gharehchopogh (2022) enhanced the exploration ability of the ICA with artificial bee colony optimization so as to enhance the global optimization ability. Li, Su & Lei (2021) added the concept of national cooperation into the ICA and used the improved algorithm to solve the scheduling optimization problems. ICA is also used in structural damage detection (Gerist & Maheri, 2019). Subsequent improvements to the ICA have also continued, Li, Lei & Cai (2019) developed a two-level ICA (TICA) based on ICA and applied it to optimize hybrid-flow shop scheduling problems. Barkhoda & Sheikhi (2020) introduced immigration concepts to ICA (IICA), and used the improved IICA to optimize the layout of wireless sensor networks. It can be concluded from the above, when it comes to optimization problems, ICA opens up a new way of thinking for researchers in addition to the commonly used solutions such as PSO and GA.

The original ICA has good exploitation ability by virtue of the competition and elimination mechanism within and between empires, while the exploration ability of the algorithm may be relatively weak. Therefore, ICA has the risk of premature convergence and falling into local optimal values. Most of the above-mentioned algorithm improvements revolve around improving the exploration ability of the ICA and enhancing the diversity of solution sets, enhancing the global optimization capability of the algorithm. After improving the exploration ability of ICA, the exploration ability and exploitation ability of ICA will be more balanced, and it can try to avoid falling into the local optimal solution too early.

In order to improve the shortcomings of ICA in global optimization, this article also proposes a new and improved idea from the perspective of improving the exploration capability, pursuing to find the global optimal solution with higher search accuracy. The original ICA enhanced country diversity through use of an assimilation step, but it simply moved at a fixed step size and angle, which limited the randomness of the countries. The assimilation step in this paper is changed into one whereby colonial countries approach an imperialist country in a spiral rising way, so as to achieve better enhanced global search ability. As the movement mode close to the optimal value of colonial countries is optimized, the convergence speed of the optimization algorithm can also be improved to some extent.

The improved ICA is named the Spiral Rising Imperialist Competitive Algorithm (SR-ICA). Herein, we not only discuss the superiority of the improved algorithm theoretically, but also test its performance on many benchmark functions and evaluate it using a variety of methods.

The rest of the article is organized as follows: Section 2 reviews the original ICA and other knowledge related to the improved algorithm. Section 3 introduces the improved algorithm and describes the implementation thereof. Sections 4 and 5 verify the performance of the improved ICA through several experiments and apply the improved SR-ICA optimization algorithm to a path planning problem to observe its optimization ability in robot path planning application. Finally, Section 6 concludes.

## Related Work

### Imperialist competitive algorithm

The ICA is a stochastic optimization search method inspired by societal interactions, which simulates the process of imperialist invasion and colonization. The original ICA consists of four parts: the creation of the initial empires, the assimilation mechanism, the competition mechanism, and the collapse of the empires.

The individual population in the ICA is a country, which is equivalent to each particle in the PSO. In solving n-dimensional problems, the state can be defined as follows:

(1) \begin{eqnarray*}country=[{p}_{1},{p}_{2},\ldots ,{p}_{N}].\end{eqnarray*}

A cost function is then used to describe the power of each country, which will affect the division between imperialist countries and colonial countries.

(2) \begin{eqnarray*}cost=f \left( country \right) =f({p}_{1},{p}_{2},\ldots ,{p}_{N}).\end{eqnarray*}

The way to measure the power of a country through the cost function is as follows: the smaller the value of the cost function, the greater the power of a country. The specific steps of this division of empires are as follows: *N*_*pop*_ countries are randomly generated, and *N*_*imp*_ imperialist countries and *N*_*col*_ colonial countries are divided according to the power of the countries. In the process of forming empires, colonies are also distributed according to the power of imperialist countries. The number of colonies in each empire is distributed as follows:

(3) \begin{eqnarray*}{C}_{n}={c}_{n}-{i}^{max} \left\{ {c}_{i} \right\} \end{eqnarray*}

(4) \begin{eqnarray*}{p}_{n}= \left\vert \frac{{C}_{n}}{\sum _{i=1}^{{N}_{imp}}{C}_{i}} \right\vert \end{eqnarray*}

(5) \begin{eqnarray*}N.C{.}_{n}=round\{ {p}_{n}\times {N}_{col}\} \end{eqnarray*}

where, *c*_*n*_ is the cost of the nth imperialist country and *C*_*n*_ is its normalized cost. *N*.*C*._*n*_ represents the number of colonies of empire *n*, *p*_*n*_ is the standardized size of that empire.

This procedure randomly assigns the remaining colonies to each imperialist country according to the number of colonies assigned to each empire, thus forming *N*_*imp*_ initial empires.

After the imperialist countries occupy their colonies, the better to rule their colonies, the imperialist countries will completely control the colonial countries from political, economic, cultural, and other aspects of assimilation, which gives rise to the assimilation mechanism in the algorithm. The ICA simulates this idea through the process of the movement of the colonial countries towards the imperialist countries, details as follows.

The step size of a colony moving to its imperialist country is defined as *x*:

(6) \begin{eqnarray*}x\sim U(0,\beta \times d)\end{eqnarray*}

*β* > 1, *d* is the distance between an imperialist country and its colonies. To increase the search space and enable the colonies to find better positions, the offset direction *θ* is added in the process of moving closer to the colonies, the value of *θ* is shown in Formula (7).

(7) \begin{eqnarray*}\theta \sim U(-\gamma ,\gamma )\end{eqnarray*}

where *γ* ∈ (0, *π*),the added offset *θ* is used to increase the population diversity.

Colonies realize their assimilation mechanism through such movement. In the process of moving towards imperialist countries, colonies may find better geographical positions and thus accrue greater national power. If the power of the colonial countries exceeds that of the imperialist countries, then there will be inter-imperial competition, and the colonial countries with greater power will replace the imperialist countries at the present stage and become the new ruling leader, while the original imperialist countries become its colonies, thus realizing the internal replacement of the empire.

In addition to competition within empires, there is also competition between empires. The imperial competition mechanism is based on the blueprint that the more powerful empires in reality will have more desire and ability to control, and the powerful empires will colonize and re-expand. This idea is reflected in ICA as follows:

First, we need to calculate the total value of each empire and determine the total power of each empire. In this process, it is stipulated that the imperialist countries exert greater influence on the total balance of power, while the colonies have a smaller influence on the total power of the empire. In the ICA, Eq. (8) is adopted to calculate the total cost of an empire,

(8) \begin{eqnarray*}T.C{.}_{n}=f \left( im{p}_{n} \right) +\xi \times \frac{\sum _{i=1}^{N.C{.}_{n}}f(co{l}_{i})}{N.C{.}_{n}} \end{eqnarray*}

where, *imp*_*n*_ is the *n*th imperialist country, *T*.*C*._*n*_ represents the total cost of the nth empire, *ξ* ∈ (0, 1), the size of *ξ* determines the extent to which the colonial countries influence the total power of the whole empire.

After calculating the total power of the empires, we compare data and find the weakest empire: this will be invaded by the stronger empires. The weakest colonial power is chosen from the weakest empire. It would be contested by the stronger empires, and the stronger empires would be more likely to subsume the colony; because of the imperial competition mechanism, strong empires would become stronger, weak empires would lose colonies and thus cede power. When a weak empire loses all its colonies, the empire will perish, and the imperialist country will become a colony of other empires. This is the collapse of empire mechanism in ICA, and the convergence speed of ICA is accelerated due to the action of this mechanism. As the empires collapse, an empire that ideally has its imperialist country and all of its colonies in the same position is presented, at which point the algorithm stops.

### Other improvements related to ICA

ICA has been widely used due to its superior optimization performance. At the same time, researchers have improved the ICA: Nazari-Shirkouhi et al. (2010) and Atashpaz-Gargari & Lucas (2007) the authors of the original algorithm, added a Revolution step to ICA. Its purpose is to enhance the exploration ability of the algorithm and avoid premature convergence to a certain local minimum value. The executive order is to introduce the revolutionary probability factor *P*_*revolution*_ to generate random countries. If these countries are stronger than the imperialist countries, they will replace them as leaders, otherwise they will play the role of colonies. In this way, the search space is expanded and the ability to avoid falling into local optimum is enhanced. In subsequent studies, the offset direction *θ* in the assimilation step was set as a random variable (Talatahari et al., 2012), with the purpose of enhancing the exploration ability and further balancing the optimization ability of ICA.

These steps and parameter settings methods have been widely used in various ICA applications, and many researchers have conducted subsequent algorithmic improvements based thereon. The ICA with the new revolutionary step and the offset parameter *θ* set as the random variable is also called the original ICA. The original ICA and the improvement mentioned in the following article are based thereon.

Zeng et al. (2016) applied ICA to distributed energy systems under an active distribution network and proposed improvements to ICA. The improvement method of optimization and its application herein is to introduce differential evolution into ICA and they propose a differential evolution imperialist competitive algorithm (DE-ICA). This adds the differential evolution step taken by colonial countries between assimilation and imperial competition, updates the colonial countries with a certain assigned probability of differential evolution, and judges whether to replace the current colonial countries by use of a greedy strategy. The current author used this method to solve the distributed energy system scheduling optimization model, and the conclusion could effectively improve the distributed energy utilization rate and reduce overall operating costs. They also show that DE-ICA can readily solve such an optimization problem. In the fourth part of this paper, the new improved SR-ICA and DE-ICA are compared to verify the superior performance of SR-ICA in solving optimization problems.

## The Proposed ICA with an Added Concept of Spiral Rising

**Figure 1:**
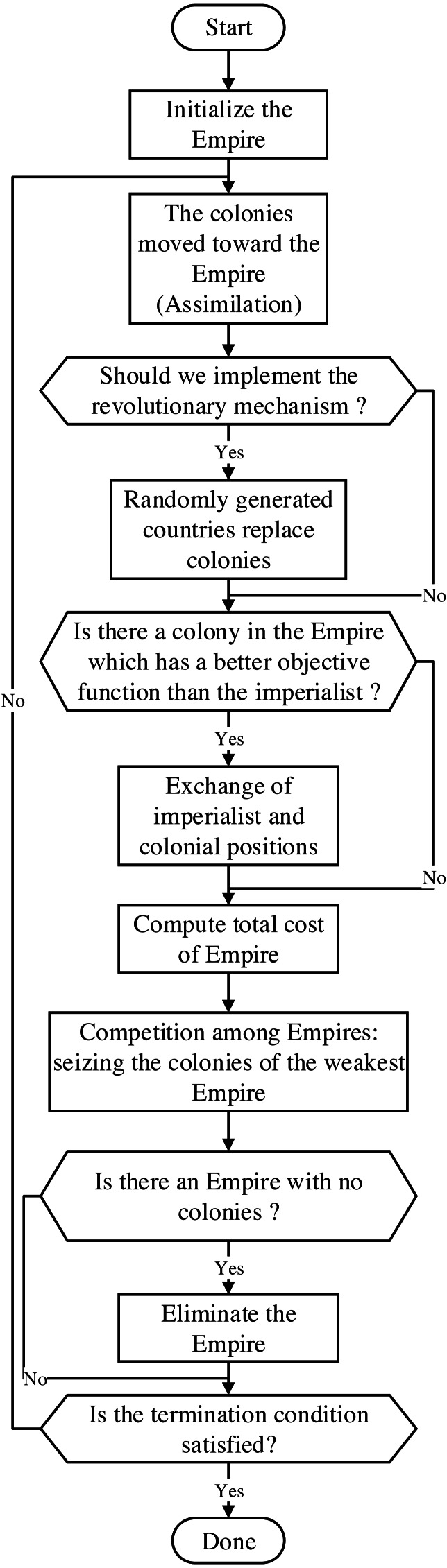
Flow-chart through the ICA.

This section describes the basic steps of the SR-ICA. Figure 1 illustrates the optimization process of the original ICA, SR-ICA is improved based on this algorithm.

As mentioned above, the imperialist countries achieved better rule over the colonies by means of cultural, political, economic and folk customs assimilation. The process of assimilation is simulated in ICA by using colonies to move in a fixed step size towards imperialist countries. However, in the theory of “spiral rise”, the general direction and trend of the development of things is a progressive movement from low to high, simple to complex, and its direction and trend always appear as “spiral rise” movement. The basic characteristics of this movement behavior are forward, circuitous and periodicity. In this paper, the assimilation step of ICA is improved by referring to the “spiral rise” theory, and the original “straight approach” movement mode of colonies is improved to “spiral rising” movement.

The whale optimization algorithm (WOA) (Mirjalili & Lewis, 2016) proposed in 2016 imitates the movement mode of humpback whales in the hunting process and approaches the global optimal value by spiral contraction, WOA has good optimization ability. The improved algorithm completely changed the assimilation mechanism in ICA by referring to the movement mode of whale hunting in WOA, and the colonies moved closer to the imperialist countries in a “spiral rising” movement mode.

**Figure 2:**
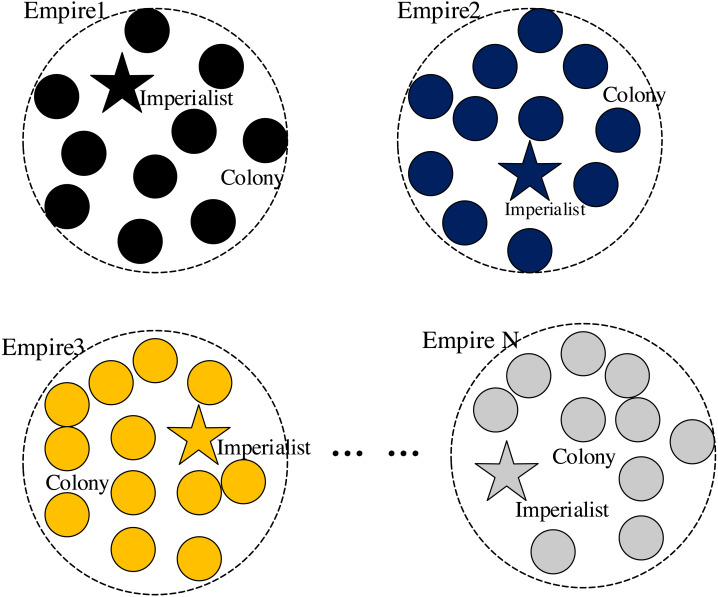
Forming the initial empires.

Figure 2 Forming the initial empires.

After the initial empires are formed, the colonial countries of each empire will move closer to the imperialist country of the empire at the center.

**Figure 3:**
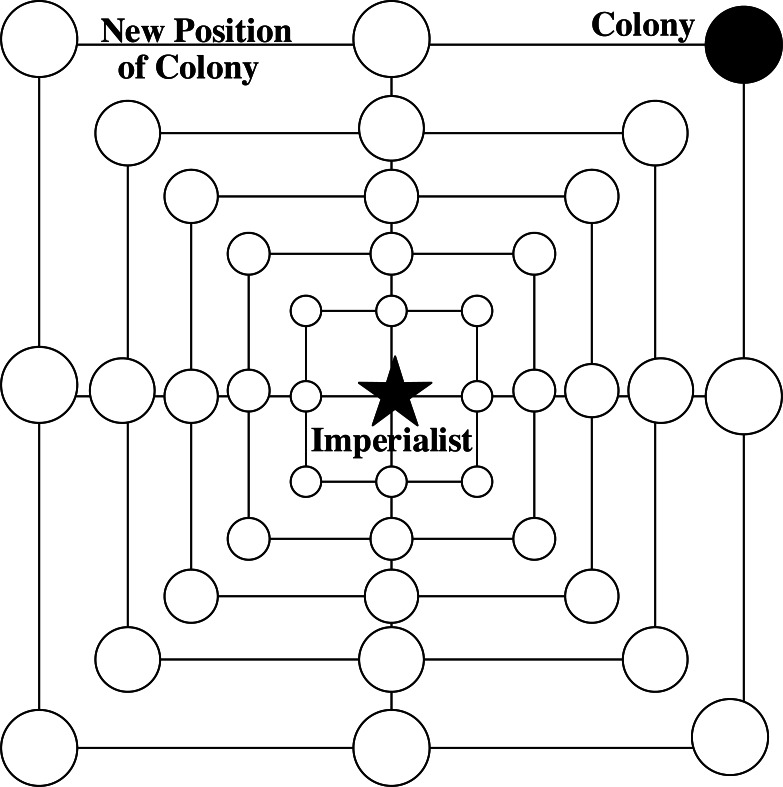
2-d diagram of SR-ICA’s shrink approach.

The whole process can be divided into two parts: (1) shrinking approach and (2) spiral circuitous approach.

**Figure 4:**
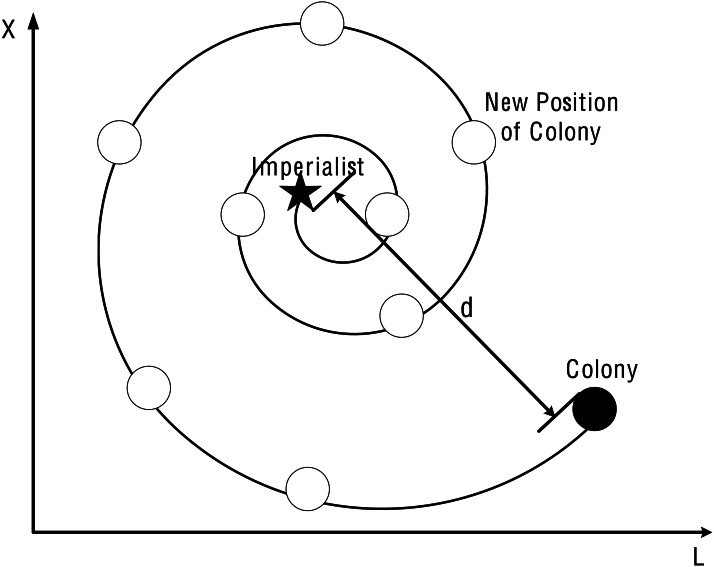
2-d diagram of SR-ICA’s spiral circuitous.

**Figure 5:**
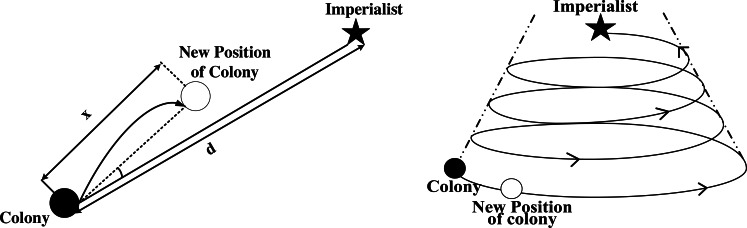
Comparison before and after assimilation steps.

(1) Shrinking approach: in a single empire, the imperialist country will be the local optimal solution, all colonies will shrink and move closer to it, and the positions of the colonies will be updated during the shrinking approach. These behaviors are represented by the following equations:
  (9) \begin{eqnarray*}\vec{D}= \left\vert \vec{C}\cdot emp \left( k \right) .imp.pos \left( t \right) -emp \left( k \right) .col \left( i \right) .pos(t) \right\vert \end{eqnarray*}
  (10) \begin{eqnarray*}emp \left( k \right) .col \left( i \right) .pos \left( t+1 \right) =emp \left( k \right) .imp.pos \left( t \right) -\vec{A}\cdot \vec{D}\end{eqnarray*}
  where *t* is the *t*th iteration, $\vec{A}$ and $\vec{C}$ are coefficient vectors, $emp \left( k \right) .imp.pos$ refers to the position vector of the imperialist country of the *k*th empire, $emp \left( k \right) .col \left( i \right) .pos$ is the position vector of the *i*th colonial country of the *k*th empire.
  Coefficient vectors $\vec{A}$ and $\vec{C}$ are given by:
  (11) \begin{eqnarray*}\vec{A}=2\vec{a}\cdot \vec{r}-\vec{a}\end{eqnarray*}
  (12) \begin{eqnarray*}\vec{C}=2\cdot \vec{r}\end{eqnarray*}
  where, $\vec{a}$ linearly decreases from 2 to 0 during the iterative process, and $\vec{r}$ is a random vector on [0,1].
  The shrink approach is achieved through the reduction of $\vec{a}$, because the change in $\vec{A}$ is related to $\vec{a}$. In this process, $\vec{A}$ is a random value on [-a, a], so the change in $\vec{A}$ is affected by $\vec{a}$. When $\vec{A}$ takes a random value on [−1,1], it means that the movement strategy of the colonial countries is anywhere between the current positions and the position of their imperial power. Figure 3 shows some of the positions to which a colony might move in a 2-d space, as achieved by changing $\vec{A}$.
(2) Spiral circuitous approach: in a single empire, the second strategy for colonial countries to approach the imperial power is termed spiral circuitous, as given by:
  (13) \begin{eqnarray*}\vec{\dot {D}}= \left\vert emp \left( k \right) .imp.pos \left( t \right) -emp \left( k \right) .col \left( i \right) .pos(t) \right\vert \end{eqnarray*}
  (14) \begin{eqnarray*}emp \left( k \right) .col \left( i \right) .pos \left( t+1 \right) =\vec{\dot {D}}\cdot {e}^{bl}\cdot \mathit{cos} \left( 2\pi l \right) +emp \left( k \right) .imp.pos(t)\end{eqnarray*}
  Where *b* is a constant for defining the shape of the logarithmic spiral; *l* is a random number on [−1,1]. Figure 4 2-d diagram of SR-ICA’s spiral circuitous.
  The addition of spiral rising mechanism can increase the spatial diversity of colonies, so in the whole process of assimilation, the positional changes are governed (among colonial countries) by the spiral rising mechanism. To synchronize the two movements, the position update factor *p* is introduced and the probability is set to 0.5. The mathematical model of this synchronization behavior is as follows:
  (15) \begin{eqnarray*}emp \left( k \right) .col \left( i \right) .pos \left( t+1 \right) = \left\{ \begin{array}{@{}ll@{}} \displaystyle emp \left( k \right) .imp.pos \left( t \right) -\vec{A}\cdot \vec{D}, &\displaystyle p\lt 0.5\\ \displaystyle \vec{\dot {D}}\cdot {e}^{bl}\cdot \mathit{cos} \left( 2\pi l \right) +emp \left( k \right) .imp.pos(t), &\displaystyle p\geq 0.5 \end{array} \right. \end{eqnarray*}
  The value of *p* is a random value on [0,1], $\vec{D}$ and $\vec{\dot {D}}$ are the distances between countries with two different modes of motion.
  The following Fig. 5 shows the comparisonof assimilation step before and after improvement.
  The spiral rising path is a zig-zag and round-about way, which approaches the global optimal solution in a special cyclic way. In the process of assimilation, the approach of colonial countries to imperialist countries in this way is conducive to increasing the optimal search space; on the premise of ensuring the enhancement of exploration ability, the exploitation ability will not be weakened, and the rate convergence will not decrease. The algorithm structure of the improved algorithm SR-ICA is presented in Table 1.

## Experimental Verification and Comparisons

Various experiments are designed to verify the optimized performance of the proposed SR-ICA. First, SR-ICA is compared with its homologous optimization algorithms, the solution accuracy and rate of convergence of SR-ICA are evaluated by benchmark functions. Then the statistical significance tests and the complexity analysis of calculation time are carried out. In addition, the improved algorithm is compared with some other commonly used advanced optimization algorithms, and the performance of the algorithms is analyzed according to the experimental results. We also apply SR-ICA to solve high-dimensional problems, observing whether it can maintain its optimization ability in the face of high-dimensional problems, finally this paper will use SR-ICA to solve the path planning problem, proposing a new solution to the path planning problems.

**Table 1:** SR-ICA algorithm structure framework.

Steps	Content
Step 1: Forming the initial empire	Countries are generated according to Eq. (1), and the generation value of each country is calculated according to Eq. (2). Countries are divided into imperialist countries and colonial countries according to Eqs. (3), Eqs. (4) and Eqs. (5), and colonial countries are assigned to form the initial empire.
Step 2: Assimilation	According to Eqs. (15), the colonial countries move closer to the imperialist countries in two different ways, the assimilation step is executed, and the position of the colonial countries is updated.
Step 3: Revolution	Some new countries are randomly generated to replace the existing colonial countries and realize the renewal of colonial countries.
Step 4: Empire internal competition	If the power of a colonial country exceeds that of the imperialist state to which it belongs, it will replace it as a new imperialist state.
Step 5: Empire external competition	Through formula (8), find out the weakest empire, its colony will be divided by other powers.
Step 6: Eliminate the empire	If there is an empire without a colony, the imperialist country will become a colony of other empires and the empire will be eliminated.

### Benchmark functions

**Figure 6:**
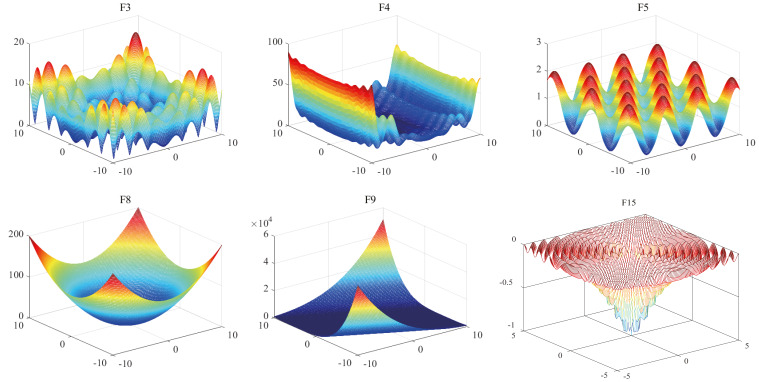
3-D image of benchmark functions.

Benchmark functions are generally used as a benchmark when evaluating the performance of intelligent optimization algorithms (Zhang et al., 2020). Different benchmark functions can be used to investigate the ability of intelligent optimization algorithms to deal with problems in different optimization situations. To measure the optimization ability of SR-ICA, 19 benchmark functions are selected (Table 2, Fig. 6).

**Table 2:** Benchmark functions.

Group	Test function	Dim	Search range	fmin	Name
Multimodal	${f}_{1} \left( x \right) =-20\exp \left( -0.2\sqrt{ \frac{1}{d} {\mathop{\sum }\nolimits }_{i=1}^{d}{x}_{i}^{2}} \right) -\exp \left( \frac{1}{d} {\mathop{\sum }\nolimits }_{i=1}^{d}\cos \left( 2\pi {x}_{i} \right) \right) +20+\exp \left( 1 \right) $	30	[−32,32]	0	Ackley
	${f}_{\mathrm{2}} \left( x \right) =10d+{\mathop{\sum }\nolimits }_{i=1}^{d} \left[ {x}_{i}^{2}-10\cos \left( 2\pi {x}_{i} \right) \right] $	30	[−10,10]	0	Rastrigin
	${f}_{\mathrm{3}} \left( x \right) ={\mathop{\sum }\nolimits }_{i=1}^{d} \left\vert {x}_{i}\sin \left( {x}_{i} \right) +0.1{x}_{i} \right\vert $	30	[−10,10]	0	Alpine
	${f}_{\mathrm{4}} \left( x \right) ={\sin }^{2}(\pi {w}_{1})+{\mathop{\sum }\nolimits }_{i=1}^{d-1}{ \left( {w}_{i}-1 \right) }^{2} \left[ 1+10{\sin }^{2} \left( \pi {w}_{i}+1 \right) \right] +{ \left( {w}_{d}-1 \right) }^{2} \left[ 1+{\sin }^{2} \left( 2\pi {w}_{d} \right) \right] $ ${w}_{i}=1+ \frac{{x}_{i}-1}{4} $	30	[−10,10]	0	Levy
	${f}_{\mathrm{5}} \left( x \right) ={\mathop{\sum }\nolimits }_{i=1}^{d} \frac{{x}_{i}^{2}}{4000} -{\Pi }_{i=1}^{d}\cos \left( \frac{{x}_{i}}{\sqrt{i}} \right) +1$	30	[−600,600]	0	Griewank
	${f}_{\mathrm{6}}(x)={\mathop{\sum }\nolimits }_{i=1}^{d} \left\vert {x}_{i} \right\vert +{\mathop{\prod }\nolimits }_{i=1}^{d} \left\vert {x}_{i} \right\vert $	30	[−10,10]	0	Schwefe2.l22
	$\begin{array}{@{}l@{}} \displaystyle {f}_{\mathrm{7}}(x)=0.1\cdot {\sin }^{2}(3\pi {x}_{1})+{\mathop{\sum }\nolimits }_{i=1}^{d}({x}_{i}-1)^{2}[1+{\sin }^{2}(3\pi {x}_{i+1})]\\ \displaystyle +({x}_{d}-1)^{2}\cdot [1+{\sin }^{2}(2\pi {x}_{d})] \end{array}$	30	[−5,5]	0	levy_montalo
Unimodal	${f}_{\mathrm{8}} \left( x \right) ={\mathop{\sum }\nolimits }_{i=1}^{d}{x}_{i}^{2}$	30	[−100,100]	0	Sphere
	${f}_{\mathrm{9}} \left( x \right) ={\mathop{\sum }\nolimits }_{i=1}^{d}{x}_{i}^{2}+{ \left( {\mathop{\sum }\nolimits }_{i=1}^{d}0.5i{x}_{i} \right) }^{2}+{ \left( {\mathop{\sum }\nolimits }_{i=1}^{d}0.5i{x}_{i} \right) }^{4}$	30	[−5,10]	0	Zakharove
	${f}_{\mathrm{10}}(x)={\mathop{\sum }\nolimits }_{i=1}^{d}[100({x}_{i+1}-{x}_{i}^{2})^{2}+({x}_{i}-1)^{2}]$	30	[−5,10]	0	Rosenbrock
	${f}_{\mathrm{11}} \left( x \right) ={ \left( {x}_{1}-1 \right) }^{2}+{\mathop{\sum }\nolimits }_{i=2}^{d}i{ \left( 2{x}_{i}^{2}-{x}_{i-1} \right) }^{2}$	30	[−5,10]	0	Dixonprice
	${f}_{\mathrm{12}}(x)={\mathop{\sum }\nolimits }_{i=1}^{d}i{x}_{i}^{2}$	30	[−10,10]	0	Sumsquare
	${f}_{\mathrm{13}}(x)={\mathop{\sum }\nolimits }_{i=1}^{d}i{x}_{i}^{4}+random[0,1)$	30	[−1.28,1.28]	0	Quartic
	${f}_{\mathrm{14}}(x)={\mathop{\sum }\nolimits }_{i=1}^{d}(1{0}^{6})^{ \frac{i-1}{d-1} }{x}_{i}^{2}$	30	[−100,100]	0	Elliptic
Fixed low dimensional	${f}_{\mathrm{15}}(x)=- \frac{1+\cos (12\sqrt{{x}_{1}^{2}+{x}_{2}^{2}})}{0.5({x}_{1}^{2}+{x}_{2}^{2})+2} $	2	[−5.12,5.12]	−1	Drop_wave
	${f}_{\mathrm{16}}(x)=0.5+ \frac{{\sin }^{2}({x}_{1}^{2}-{x}_{2}^{2})-0.5}{[1+0.001({x}_{1}^{2}+{x}_{2}^{2})]^{2}} $	2	[−100,100]	0	Schaffer
	${f}_{\mathrm{17}}(x)=-0.0001( \left\vert \sin ({x}_{1})\sin ({x}_{2})\exp ( \left\vert 100- \frac{\sqrt{{x}_{1}^{2}+{x}_{2}^{2}}}{\pi } \right\vert ) \right\vert +1)^{0.1}$	2	[−10,10]	−2.06	Cross_in_tray
	${f}_{18}(x)=0.26({x}_{1}^{2}+{x}_{2}^{2})-0.48{x}_{1}{x}_{2}$	2	[−10,10]	0	Matyas
	*f*_19_(*x*) = − cos(*x*_1_)cos(*x*_2_)exp(−(*x*_1_ − *π*)^2^ − (*x*_2_ − *π*)^2^)	2	[−100,100]	−1	Easom

The 19 benchmark functions can be divided into three types. The first type is multi-modal functions, which includes seven benchmark functions (F1 to F7). The benchmark functions of this type have multiple local minimum values, which can verify the exploration ability of the algorithms: because they have multiple local minima, these types of functions can be used to evaluate the ability of the optimization algorithms to escape from local optimal values. The second type are unimodal functions: of the 19 benchmark functions, seven benchmark functions (F8-F14) are of this type. They have a unique global optimal value, so there are no local optima issues arising therewith. Unimodal functions can evaluate the optimization precision and convergence speed of the optimization algorithms, and the exploitation ability of the optimization algorithms can be seen from the experimental results. The third type fixed low-dimensional functions (F15-F19) have fixed dimensions and make it easier to find the optimal value than the first two benchmark functions. The dimension settings, search ranges, and global minima of these baseline functions are detailed in Table 2.

### Comparison with homologous optimization algorithms

In this part, ICA, WOA and the newly improved SR-ICA were compared to prove that the new method represents an improvement over WOA and ICA in terms of optimization performance. SR-ICA was also compared to the DE-ICA (mentioned in Section 2). These four intelligent optimization algorithms were used to solve the aforementioned 19 benchmark functions, and then the experimental data obtained are compared and analyzed.

In order to better compare the performance of four different intelligent optimization algorithms and reduce the impact of randomness and parameter selection on optimization search results, ICA, DE-ICA and WOA algorithms were set as optimal parameters by referring to relevant literatures. SR-ICA proposed in this article set each parameter to the optimal value by controlling variables. The specific parameter setting data are shown in Table 3.

**Table 3:** Algorithm parameter settings.

Algorithm	Parameter settings
ICA	nPop=200; nEmp=3; alpha=1; beta=2; zeta=0.1; MaxIt=300 pRevolution=0.05; mu=0.1
WOA	*N* = 200; MaxIt=300; *e* = 2.717; *b* = 1; *P* = 0.5
DE-ICA	nPop=200; nEmp=3; alpha=1; beta=2; zeta=0.1; MaxIt=300 pRevolution=0.05; mu=0.1; *F* = 0.6; CR=0.9
SR-ICA	nPop=200; nEmp=3; alpha=1; zeta=0.1; MaxIt=300 pRevolution=0.05; mu=0.1; *e* = 2.717; *b* = 5; *P* = 0.5

The total population (in each of these four optimization algorithms) was set to 200, and 300 number iterations were used. To reduce the effect of randomness on the results and subsequent evaluation, we run the optimization process of each benchmark function 30 times, and take the average value as the final result. The optimization results arising from use of the four intelligent optimization algorithms on the 19 benchmark functions are shown in Table 4 to Table 6.

**Table 4:** Experimental results: multi-modal functions.

Test function	ICA	WOA	DE-ICA	SR-ICA
F1	Ave:2.03E−04 Std:2.39E−04	Ave:5.03E−15 Std:2.65E−15	Ave:1.49E−05 Std:1.50E−05	Ave:8.88E−16 Std:8.88E−16
F2	Ave:1.34E+01 Std:3.66E+00	Ave:0.00E+00 Std:0.00E+00	Ave:1.15E+01 Std:3.98E+00	Ave:0.00E+00 Std:0.00E+00
F3	Ave:7.58E−06 Std:1.04E−05	Ave:4.32E−01 Std:2.36E+00	Ave:1.67E−06 Std:2.01E−06	Ave:8.78E−13 Std:4.81E−12
F4	Ave:2.46E−07 Std:1.14E−06	Ave:2.42E−02 Std:4.56E−02	Ave:6.54E−09 Std:2.11E−08	Ave:5.16E−14 Std:1.64E−13
F5	Ave:2.29E−02 Std:2.45E−02	Ave:1.67E−03 Std:9.14E−03	Ave:1.49E−02 Std:2.03E−02	Ave:0.00E+00 Std:0.00E+00
F6	Ave:3.73E−06 Std:3.96E−06	Ave:3.11E−37 Std:5.41E−37	Ave:1.77E−07 Std:1.31E−07	Ave:3.34E−182 Std:0.00E+00
F7	Ave:1.77E−10 Std:3.37E−10	Ave:1.68E−02 Std:2.12E−02	Ave:3.28E−12 Std:3.99E−12	Ave:6.69E−14 Std:2.74E−13

**Table 5:** Experimental results: unimodal functions.

Test function	ICA	WOA	DE-ICA	SR-ICA
F8	Ave:5.34E−08 Std:5.47E−08	Ave:4.13E−62 Std:2.25E−61	Ave:4.80E−10 Std:5.92E−10	Ave:0.00E+00 Std:0.00E+00
F9	Ave:2.80E+00 Std:1.75E+00	Ave:4.61E+02 Std:8.51E+01	Ave:1.19E−02 Std:9.38E−03	Ave:7.71E−87 Std:4.22E−86
F10	Ave:4.75E+01 Std:4.15E+01	Ave:2.58E+01 Std:4.84E+00	Ave:4.58E+01 Std:3.38E+01	Ave:9.84E−13 Std:2.63E−12
F11	Ave:1.44E+00 Std:1.13E+00	Ave:6.67E−01 Std:2.04E−05	Ave:9.63E−01 Std:7.19E−01	Ave:2.57E−01 Std:8.51E−02
F12	Ave:9.14E−09 Std:1.33E−08	Ave:3.40E−65 Std:1.53E−64	Ave:5.74E−11 Std:8.77E−11	Ave:0.00E+00 Std:0.00E+00
F13	Ave:4.99E−02 Std:2.09E−02	Ave:8.56E−04 Std:9.49E−04	Ave:1.04E−02 Std:3.47E−03	Ave:3.03E−05 Std:2.06E−05
F14	Ave:4.87E−03 Std:6.42E−03	Ave:2.44E−59 Std:1.22E−58	Ave:6.64E−05 Std:9.01E−05	Ave:0.00E+00 Std:0.00E+00

**Table 6:** Experimental results: fixed low-dimensional test functions.

Test function	ICA	WOA	DE-ICA	SR-ICA
F15	Ave:−9.98E−01 Std:1.16E−02	Ave:−9.94E−01 Std:1.94E−02	Ave:−1.00E+00 Std:0.00E+00	Ave:−1.00E+00 Std:0.00E+00
F16	Ave:0.00E+00 Std:0.00E+00	Ave:0.00E+00 Std:0.00E+00	Ave:0.00E+00 Std:0.00E+00	Ave:0.00E+00 Std:0.00E+00
F17	Ave:−2.06E+00 Std:9.03E−16	Ave:−2.06E+00 Std:6.03E−11	Ave:−2.06E+00 Std:9.03E−16	Ave:−2.06E+00 Std:7.91E−16
F18	Ave:1.51E−38 Std:6.44E−38	Ave:9.56E−200 Std:0.00E+00	Ave:2.42E−41 Std:1.30E−40	Ave:1.44E−165 Std: 0.00E+00
F19	Ave:−1.00E+00 Std:0.00E+00	Ave:−1.00E+00 Std:1.95E−08	Ave:−1.00E+00 Std:0.00E+00	Ave:−1.00E+00 Std:6.67E−16

Table 4 shows the experimental results arising from the use of ICA, WOA, DE-ICA, and SR-ICA when applied to the solution of the first type of benchmark functions. The results show the mean values and standard deviations obtained after 30 independent runs of these four algorithms. It can be seen from the results that SR-ICA returns the best results when solving the seven benchmark functions, and F2 and F5 can even find the global optimal value. This type of benchmark function has multiple local optimal values, which indicates that SR-ICA has good exploration ability. Compared with the other three optimization algorithms, SR-ICA can better avoid local optimal values. Although F6 did not find the global minimum directly, the average result after 30 runs is very close to the minimum. Compared with DE-ICA, the optimization ability is slightly improved after the addition of differential evolution, but the effect is not very significant, while the optimization ability is improved to a certain extent after the addition of a spiral rising movement strategy.

Table 5 shows the experimental results arising from the use of the second type of benchmark functions, which are also presented by mean values and standard deviations. The values found by SR-ICA are not only the smallest among all the results, but also the global minimum value as found by SR-ICA when solving the three optimization problems of F8, F12, and F14. The second type of benchmark functions is unimodal functions, which can be used to investigate the optimization accuracy and rate of convergence of the optimization algorithms: in addition to the three benchmark functions mentioned above, good values can also be found for the other problems, especially F9, whose optimization accuracy is very high.

**Figure 7:**
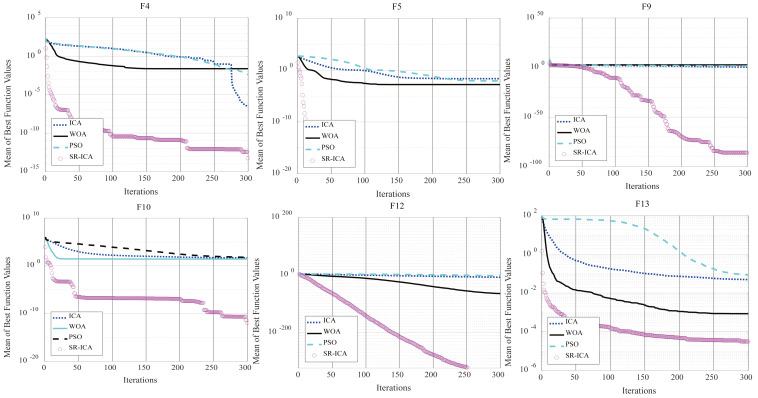
Convergence charts: four algorithms.

To observe rate of convergence of the optimization algorithms, iterative optimization convergence graphs of the first and second types of partial benchmark functions are given in Fig. 7. It can be seen from F4, F10, and F13 that SR-ICA can not only find the results with very high accuracy, but can do so rapidly. This indicates that the newly improved SR-ICA does not weaken the exploitation ability of the algorithm based on enhanced exploration ability and the convergence speed remains very fast after the addition of the spiral rising movement strategy.

Table 6 shows the optimization results arising from use in the solution of fixed low-dimensional optimization problems. Compared with the first two kinds of benchmark functions, this kind of optimization problems is easier to solve. For example, all four optimization algorithms of F16 can find optimal values. Except for F18 and F19, the results found by SR-ICA are all optimal, SR-ICA is applicable to the solution of a wider range of optimization problems.

It can be seen from the above optimization experiments that the SR-ICA intelligent optimization algorithm proposed in this article has strong global optimization capability, and can find a more accurate global optimal solution whether dealing with unimodal functions or multi-modal functions. The improved algorithm has a better exploration capability based on the inherited exploitation capability of ICA, so that the SR-ICA can better escape from the local optimal solution. Compared with other ICA improvement algorithms, SR-ICA also has the advantages of high search accuracy and faster search speed.

### Statistical significance tests of experimental results

To compare the performance of the four optimization algorithms mentioned above, a Friedman test (Pelusi et al., 2020) was used to analyze their significance in this section. A Friedman test is performed on ICA, WOA, DE-ICA, and SR-ICA at the significance level of 5%, and the test results are displayed in Table 7.

**Table 7:** Friedman test results.

Average Rank			
F1-F7		F8-F14	
Algorithm	Rank	Algorithm	Rank
ICA	3.88	ICA	3.57
WOA	2.25	WOA	2.79
DE-ICA	2.88	DE-ICA	2.57
SR-ICA	1.00	SR-ICA	1.07
*p*-value:	0.000	*p*-value:	0.003

On the left-hand side of Table 7, seven multi-modal functions are given to solve the ranking results obtained from Friedman test with four different optimization algorithms. The lower the rank value, the better the optimization performance of the algorithm. The performance of SR-ICA was the best: the *p*-value for this set of comparisons is also given below (a *p*-value of less than 0.05 indicates that these algorithms are not correlated). On the right-hand side, we show the Friedman test of four optimization algorithms for seven unimodal functions, with the *p*-value of 0.003. This result shows that SR-ICA was significantly superior to all other optimization algorithms considered for comparison in terms of solution accuracy.

The F1-F7 benchmark functions ICA and WOA were subjected to a Friedman test with SR-ICA respectively, and the *p*-values were both 0.005. F8-F14 benchmark functions ICA and WOA are tested by Friedman with SR-ICA, and the *p*-values obtained are 0.008 and 0.014, respectively. These *p*-values are less than 0.05, which verifies that SR-ICA is significantly different from ICA and WOA, and there is no correlation between the algorithms. It can also be seen from the optimization ranking results of the algorithms in Table 7 that compared with the other three algorithms, the proposed SR-ICA can achieve the best comprehensive performance.

### Time-complexity analysis

In this part, the progressive analysis of the algorithm was used to evaluate the time-complexity of the improved algorithm and compare it with ICA to observe whether the time-complexity of the algorithm increased after adding the spiral rising mechanism.

The original ICA can be divided into five parts: *T*_initialize_, *T*_assimilate_, *T*_revolution_, *T*_compete_ and *T*_total cost_. The time-complexity of this calculation is such that ICA and SR-ICA were divided into the aforementioned five parts, and the time-complexity of each part is analyzed progressively, the total time-complexity is expressed by superposition at last. Assuming that the problem dimension to be dealt with is *D*, the number of empires is *N*, the number of colonies is *n*, and the total number of iterations is *M*,the time-complexity of ICA is expressed as:

(16) \begin{eqnarray*}T \left( ICA \right) ={T}_{initialize}+ \left( {T}_{\text{assimilate}}+{T}_{\text{revolution}}+{T}_{\text{compete}}+{T}_{\text{total cost}} \right) \cdot M\nonumber\\\displaystyle = \left( N+n \right) \cdot D+ \left[ N\times n+N\times \left( N+n \right) +N\times n+N \right] \cdot M\end{eqnarray*}

After sorting, it can be found that the complexity of ICA calculation time was $T \left( ICA \right) = \left( N+n \right) \cdot D+(3n+N+1)\cdot NM$.

SR-ICA is consistent with the parameter settings of ICA when dealing with the problems, the assimilation step is changed after improvement with other parts remaining unchanged, which means that the time-complexity of the other four parts is consistent with that of ICA. The time-complexity of the assimilation step is changed to ${T}_{assimilate} \left( SR-ICA \right) =N\times n\times D$ after the improvement, therefore, the total time-complexity of SR-ICA after optimization is $T \left( SR-ICA \right) = \left( N+n \right) \cdot D+[(D+2)n+N+1]\cdot NM$. Compared with ICA, the change in the time-complexity of SR-ICA is very small. Although the cyclic calculation of dimension *D* is added to the assimilation part, the change is of the same order of magnitude, so the complexity of the calculation time of SR-ICA after improvement does not increase compared with ICA.

### Comparison with other advanced optimization algorithms

To verify the superiority of SR-ICA in solving optimization problems, in this part, we choose some commonly used advanced intelligent optimization algorithms for comparison, use these algorithms to process the above multi-modal and unimodal benchmark functions, observing the experimental results to analyze the optimization performance of SR-ICA.

In this part, we chose six types of intelligent optimization algorithms for comparative experiments. These six optimization algorithms are relatively representative among the four categories of nature-inspired meta-heuristic algorithms mentioned in the introduction (they are also intelligent optimization algorithms that are often used to solve practical optimization problems Askari, Younas & Saeed, 2020) and they are: differential evolution (DE) (Lampinen & Storn, 2004) of evolution-based optimization algorithms, particle swarm optimization (PSO), the grey wolf optimizer (GWO) (Mirjalili, Mirjalili & Lewis, 2014) of swarm-based optimization algorithms, teaching-learning based optimization (TLBO) (Rao, Savsani & Vakharia, 2011) and brain storm optimization (BSO) (El-Abd, 2017) of human behavior-based optimization algorithms, and the sine-cosine algorithm (SCA) (Mirjalili, 2016) from among the physics-based optimization algorithms.

These six representative optimization algorithms are compared with the SR-ICA proposed herein. The experimental results are shown in Tables 8 and 9. The parameters of the other six optimization algorithms are summarized in Table 10. Similarly, for the six intelligent optimization algorithms, this paper refers to the relevant literatures to set their parameters to the optimal situation.

**Table 8:** Experimental results: multi-modal benchmark functions.

F	DE	PSO	GWO	BSO	TLBO	SCA	SR-ICA
F1	Ave: 4.75E+00 Std: 6.97E−01	Ave: 4.37E−03 Std: 4.30E−03	Ave: 3.32E−14 Std: 4.79E−15	Ave: 1.40E−01 Std: 3.41E−01	Ave: 5.51E−15 Std: 1.66E−15	Ave: 1.54E+00 Std: 6.92E−01	Ave: 8.88E−16 Std: 8.88E−16
F2	Ave: 2.22E+02 Std: 1.46E+01	Ave: 4.25E+01 Std: 1.30E+01	Ave: 6.39E−01 Std: 2.60E+00	Ave: 4.68E+01 Std; 1.08E+01	Ave: 3.83E+00 Std: 4.11E+00	Ave: 6.36E+01 Std: 3.69E+01	Ave: 0.00E+00 Std: 0.00E+00
F3	Ave: 1.13E+01 Std: 1.57E+00	Ave: 5.77E−03 Std: 3.52E−03	Ave: 3.86E−04 Std: 5.10E−04	Ave: 5.08E−01 Std: 4.37E−01	Ave: 6.64E−09 Std: 3.58E−08	Ave: 6.94E−01 Std: 1.15E+00	Ave: 8.78E−13 Std: 4.81E−12
F4	Ave: 1.61E+00 Std: 5.93E−01	Ave: 3.50E−03 Std: 1.63E−02	Ave: 6.92E−01 Std: 1.59E−01	Ave: 6.04E+00 Std: 3.82E+00	Ave: 1.64E−01 Std: 1.45E−01	Ave: 2.83E+00 Std: 1.07E+00	Ave: 5.16E−14 Std: 1.64E−13
F5	Ave: 2.64E+00 Std: 7.25E−01	Ave: 8.54E−03 Std: 9.60E−03	Ave: 3.71E−04 Std: 2.03E−03	Ave: 1.27E+01 Std: 4.82E+00	Ave: 0.00E+00 Std: 0.00E+00	Ave: 1.01E+00 Std: 2.35E−01	Ave: 0.00E+00 Std: 0.00E+00
F6	Ave: 1.63E+01 Std: 5.36E+00	Ave: 6.76E−03 Std: 4.09E−03	Ave: 1.91E−19 Std: 1.68E−19	Ave: 5.51E−02 Std: 7.19E−02	Ave: 5.82E−25 Std: 1.69E−25	Ave: 6.10E−02 Std: 7.01E−02	Ave: 3.3E−182 Std: 0.00E+00
F7	Ave: 1.20E+00 Std: 9.31E−01	Ave: 3.77E−05 Std: 3.44E−05	Ave: 2.70E−01 Std: 5.10E−01	Ave: 3.33E−03 Std: 1.82E−02	Ave: 1.66E−02 Std: 3.77E−02	Ave: 1.98E+01 Std: 2.42E+00	Ave: 6.69E−14 Std: 2.74E−13

**Table 9:** Experimental results: unimodal benchmark functions.

F	DE	PSO	GWO	BSO	TLBO	SCA	SR-ICA
F8	Ave: 1.66E+02 Std: 8.52E+01	Ave: 2.82E−05 Std: 3.27E−05	Ave: 5.41E−33 Std: 7.56E−33	Ave: 8.80E−06 Std: 2.79E−06	Ave: 1.25E−49 Std: 6.40E−50	Ave: 7.28E+00 Std: 1.06E+01	Ave: 0.00E+00 Std: 0.00E+00
F9	Ave: 8.49E+00 Std: 6.29E+00	Ave: 3.59E+01 Std: 7.01E+00	Ave: 7.03E−16 Std: 1.14E−15	Ave: 8.07E+00 Std: 7.60E+00	Ave: 2.68E−03 Std: 1.81E−03	Ave: 1.27E+01 Std: 7.29E+00	Ave: 7.71E−87 Std: 4.22E−86
F10	Ave: 2.57E+02 Std: 1.04E+02	Ave: 6.04E+01 Std: 4.32E+01	Ave: 2.62E+01 Std: 6.40E−01	Ave: 5.25E+01 Std: 3.81E+01	Ave: 2.00E+01 Std: 6.30E−01	Ave: 6.76E+01 Std: 7.70E+01	Ave: 9.84E−13 Std: 2.63E−12
F11	Ave: 4.76E+01 Std: 2.59E+01	Ave: 1.32E+00 Std: 9.43E−01	Ave: 6.67E−01 Std: 1.08E−05	Ave: 1.92E+00 Std: 2.13E+00	Ave: 6.67E−01 Std: 4.79E−13	Ave: 8.59E+00 Std: 1.44E+01	Ave: 2.57E−01 Std: 8.51E−02
F12	Ave: 2.12E+01 Std: 1.00E+01	Ave: 3.57E−04 Std: 5.68E−04	Ave: 5.90E−34 Std: 8.56E−34	Ave: 1.32E−01 Std: 2.13E−01	Ave: 1.82E−50 Std: 8.84E−51	Ave: 6.32E−01 Std: 7.23E−01	Ave: 0.00E+00 Std: 0.00E+00
F13	Ave: 1.39E−01 Std: 4.07E−02	Ave: 8.70E−02 Std: 2.92E−02	Ave: 4.61E−04 Std: 2.70E−04	Ave: 4.67E−02 Std: 1.94E−02	Ave: 1.15E−03 Std: 3.75E−04	Ave: 5.47E−02 Std: 4.15E−02	Ave: 3.03E−05 Std: 2.06E−05
F14	Ave: 3.20E+05 Std: 1.27E+05	Ave: 4.26E−01 Std: 4.67E−01	Ave: 1.28E−29 Std: 2.08E−29	Ave:1.94E+06 Std: 1.59E+06	Ave: 1.84E−45 Std: 9.27E−46	Ave: 1.13E+03 Std: 1.51E+03	Ave: 0.00E+00 Std: 0.00E+00

**Table 10:** Parameter settings for optimization algorithms.

Algorithm	Parameter settings
DE	*N* = 200; MaxIt=300 *F* = 0.5; CR=0.8; mutationStrategy=1; crossStrategy=2
PSO	*N* = 200; MaxIt=300 Vmax=6; wMax=0.9; wMin=0.2; c1=2; c2=2
GWO	*N* = 200; MaxIt=300; *e* = 2.717
BSO	*N* = 200; MaxIt=300; n_c = 2; prob_one_cluster = 0.8
TLBO	*N* = 200; MaxIt=300
SCA	*N* = 200;MaxIt=300; *a* = 2
SR-ICA	nPop=200; nEmp=3; alpha=1; zeta=0.1; MaxIt=300 pRevolution=0.05; mu=0.1; *e* = 2.717; *b* = 5; *P* = 0.5

Compared with the six optimization algorithms mentioned above, the minimum values found by SR-ICA in the optimization problems of the 14 benchmark functions are the best. The five benchmark functions F2, F5, F8, F12, and F14 can all find their minimum values using SR-ICA, among which only the F5 benchmark function TLBO can also find its minimum value.

**Figure 8:**
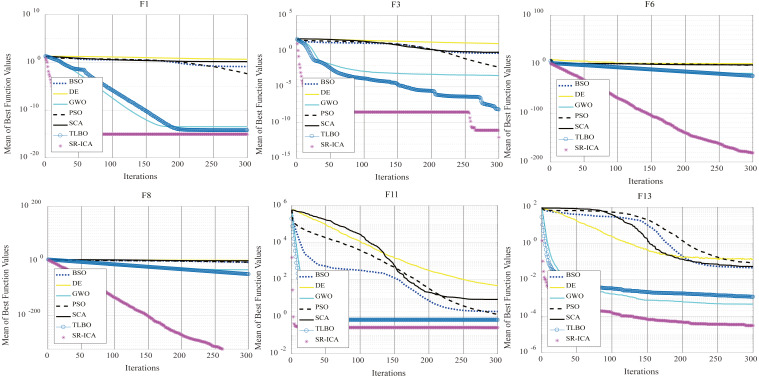
Convergence charts: seven algorithms.

In view of the experimental data from the seven optimization algorithms and the convergence of each optimization process (Fig. 8), SR-ICA has high convergence precision and a rapid rate of convergence. The optimization performance of the improved algorithm is assessed from different angles by unimodal and multi-modal functions. After adding the spiral-rise mechanism, the population diversity of the original ICA is improved, and its exploration ability is greatly enhanced. Compared with other commonly used advanced intelligent optimization algorithms, the ability of SR-ICA to solve optimization problems is also very good.

### Ability to solve high-dimensional problems

When the dimension of optimization problems increases, the complexity of solution also increases. Such large-scale problems are more common in engineering practice. Large-scale optimization problems generally mean that the number of dimensions in the optimization problem exceeds 100. When dealing with such high-dimensional problems, some intelligent optimization algorithms may lose their usefulness and fail to find a good global optimal solution, resulting in the so-called “dimension disaster” problem (Aguilar-Justo & Mezura-Montes, 2019).

To investigate the ability of SR-ICA to solve large-scale optimization problems, six benchmark functions, F1, F2, F5, F8, F12, and F14, were selected from the 19 benchmark functions involved in the experiment to conduct high-dimensional optimization trials on the optimization algorithms. We not only chose ICA and WOA for experimental comparison, but also chose PSO and TLBO, which have strong optimization ability as evinced by the findings in the previous section, for SR-ICA comparative experiments, we observed the experimental results and analyze the performance of SR-ICA in dealing with large-scale optimization problems. The experimental results are presented in Table 11.

**Table 11:** Experimental results: high dimensional optimization problems.

Test function	Dim	PSO	WOA	ICA	TLBO	SR-ICA
F1	300	Ave:7.72E+00 Std:2.70E−01	Ave:4.44E−15 Std:2.29E−15	Ave:1.96E+01 Std:4.94E−02	Ave:6.07E−02 Std:3.33E−01	Ave:8.88E−16 Std: 0.00E+00
	500	Ave:1.05E+01 Std: 2.17E−01	Ave:4.20E−15 Std: 2.07E−15	Ave:1.98E+01 Std:4.15E−02	Ave:7.68E−01 Std:1.11E+00	Ave:8.88E−16 Std: 0.00E+00
F2	300	Ave:3.87E+03 Std:2.19E+02	Ave:3.03E−14 Std:1.15E−13	Ave:5.96E+03 Std:3.34E+02	Ave:0.00E+00 Std:0.00E+00	Ave:0.00E+00 Std:0.00E+00
	500	Ave:8.66E+03 Std:4.28E+02	Ave:3.03E−14 Std:1.66E−13	Ave:1.24E+04 Std: 5.60E+02	Ave:0.00E+00 Std:0.00E+00	Ave:0.00E+00 Std:0.00E+00
F5	300	Ave:1.33E+01 Std:2.45E+00	Ave:0.00E+00 Std:0.00E+00	Ave:1.26E+02 Std:1.38E+01	Ave:0.00E+00 Std:0.00E+00	Ave:0.00E+00 Std:0.00E+00
	500	Ave:4.04E+01 Std:4.86E+00	Ave:0.00E+00 Std:0.00E+00	Ave:1.16E+03 Std:9.74E+01	Ave:1.11E−16 Std:0.00E+00	Ave:0.00E+00 Std:0.00E+00
F8	300	Ave:8.97E+02 Std:9.17E+01	Ave:2.58E−60 Std:8.97E−60	Ave:1.38E+04 Std: 1.51E+03	Ave:7.80E−41 Std:3.00E−41	Ave:0.00E+00 Std:0.00E+00
	500	Ave:4.08E+03 Std:2.01E+02	Ave:2.39E−60 Std:1.18E−59	Ave:1.26E+05 Std:1.48E+04	Ave:2.72E−40 Std:9.89E−41	Ave:0.00E+00 Std:0.00E+00
F12	300	Ave:9.80E+04 Std:1.14E+04	Ave:1.57E−61 Std:4.30E−61	Ave:2.93E+04 Std:5.16E+03	Ave:6.28E−41 Std:1.71E−41	Ave:0.00E+00 Std:0.00E+00
	500	Ave:5.84E+05 Std:2.53E+04	Ave:8.28E−61 Std:1.58E−60	Ave:3.08E+05 Std:3.17E+04	Ave:6.29E−40 Std:1.56E−40	Ave:0.00E+00 Std:0.00E+00
F14	300	Ave:2.64E+07 Std:5.20E+06	Ave:4.34E−55 Std:2.36E−54	Ave:2.16E+08 Std:4.82E+07	Ave:1.22E−36 Std:5.23E−37	Ave:0.00E+00 Std:0.00E+00
	500	Ave:1.26E+08 Std: 2.29E+07	Ave:3.95E−56 Std:1.34E−55	Ave:1.42E+09 Std:2.06E+08	Ave:6.04E−36 Std:3.23E−36	Ave:0.00E+00 Std:0.00E+00

When testing the processing ability of the optimization algorithms for high-dimensional problems, the numbers of dimensions of the six benchmark functions are set to 300 and 500. Parameter settings of the five optimization algorithms all refer to the optimal settings mentioned above. Each optimization problem is run independently some 30 times, and the experimental results are averaged.

It can be seen from the experimental data in the above table that PSO and ICA are not capable of solving high-dimensional optimization problems. When the number of dimensions is set to 300 and 500, the global optimization ability of PSO and ICA is significantly weakened, and the optimization results of some functions have completely deviated from the global minimum values. In contrast, when WOA and TLBO are used on such problems, the optimization results are not greatly affected, and the global optimal values remain close to the minimum values of the function. WOA, when solving F5, and TLBO when solving F2 and F5, can even find the minimum values. When the number of dimensions increases from 300 to 500, the optimization ability of WOA and TLBO is not affected to any significant extent.

As can be seen from the experimental results of SR-ICA, it inherits the excellent ability of WOA to solve high-dimensional problems in the process of improvement of optimization algorithm, and its optimization ability is not greatly affected when the number of dimensions is greatly increased. SR-ICA can still find their global minimum values when processing F2, F5, F8, F12, and F14, and the experimental results of F1 are also consistent with the experimental results of 30 dimensions. SR-ICA not only further balances the exploitation and exploration capabilities, but also changes the search space movement strategy of the original ICA. In the face of large-scale problems with high complexity, SR-ICA can also provide good search results by relying on its advantage of spatial movement.

## Optimization Analysis of Path Planning Problems

In this section, the SR-ICA was applied to the mobile robot path planning problem (MRPPP), and the path optimization comparison with other optimization algorithms is made to analyze the ability of SR-ICA method to find the optimal path.

In this experiment, MRPPP refers to finding a path with the shortest distance from a given starting point to a given end point that can avoid all obstacles in the area. This is an important technique used in design of mobile robots (Deng et al., 2021). The practical application of the optimization algorithms is to choose the path planning problems, use SR-ICA and other optimization algorithms to find the optimal path of robot movement.

**Figure 9:**
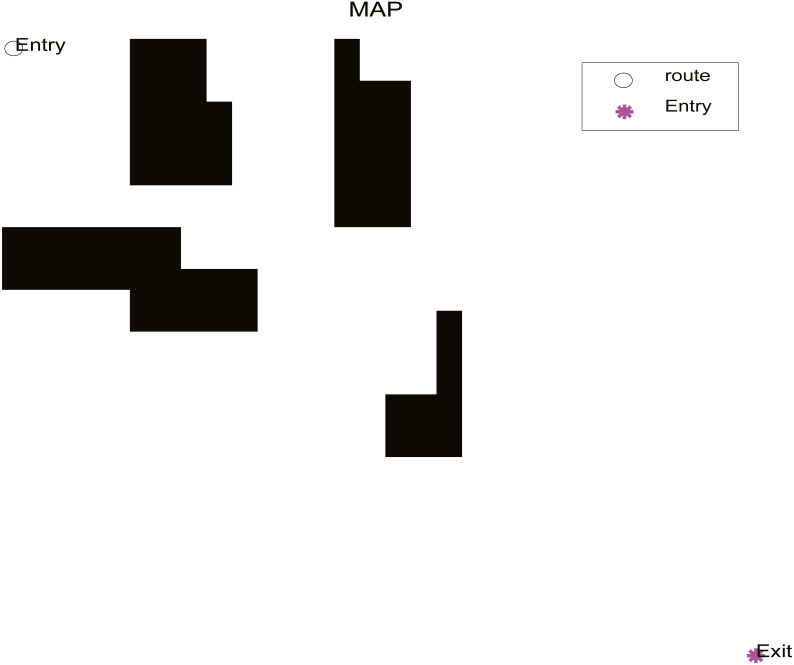
Obstacle distribution.

This simulation experiment was an off-line path planning task, and the planning environment was fixed, which is known as static environment planning. Obstacles are set in advance in the known global static 30 × 30 raster matrix environment, and the starting coordinates of the robot are (1,1), and the end coordinates are (30,30). The distribution of obstacles is shown in Fig. 9.

When finding the optimal path, our requirement is to find the path with the shortest distance without touching obstacles, and to apply the optimization algorithms to finding the shortest path. In this way, the objective function of the optimization algorithms is as shown in Eq. (17):

(17) \begin{eqnarray*}L \left( Path \right) =\sum _{i=0}^{n-1}\sqrt{(x{l}_{i+1}-x{l}_{i})^{2}+(y{l}_{i+1}-y{l}_{i})^{2}}.\end{eqnarray*}

**Figure 10:**
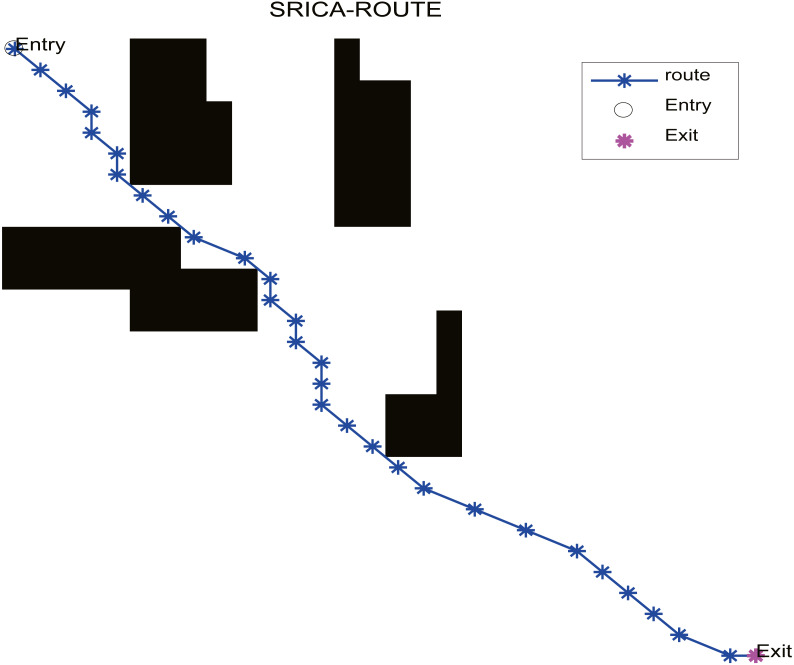
SR-ICA path optimization.

In the practical application, we select the optimization algorithm SR-ICA for optimal path optimization, and also choose ICA, WOA, and PSO for purposes of comparison. When using these four optimization algorithms to find the optimal paths, if the path found fails to avoid all of the obstacles, we will regard it as having failed to find the optimal path. SR-ICA optimization of the path problem is made, the results of solving the above path planning problem are shown in Fig. 10.

**Figure 11:**
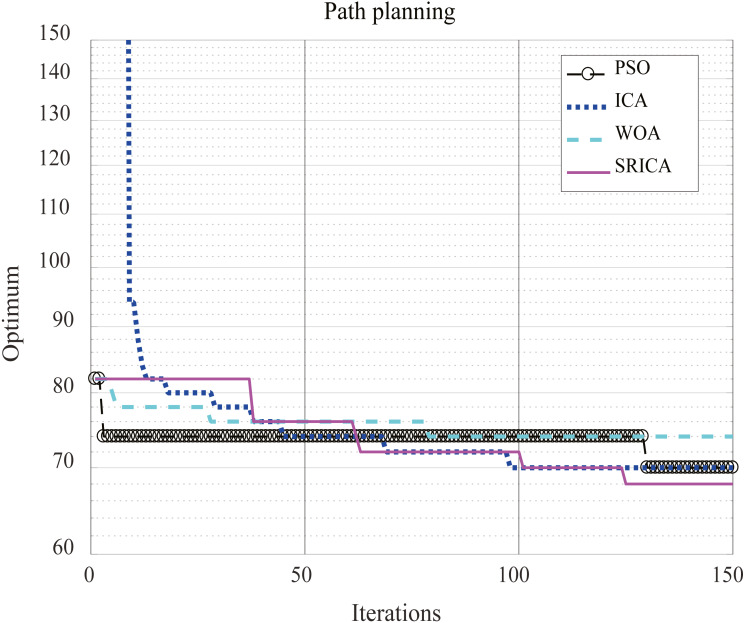
Comparison of path planning results.

The results of the four optimization algorithms used to solve the above path planning problem are listed in Table 12, and the optimization performance comparison results are shown in Fig. 11. The parameter settings of these four optimization algorithms when solving the problem are summarized in Table 13. The population was set to 100 when using these four optimization algorithms and the number of iterations was set to 150.

According to the path distance results obtained by the different methods used to solve the path planning problem of the set off-line single robot in Table 12, it can be seen that the optimized path result distance of SR-ICA is the shortest, indicating that this optimization method can find a more optimized effective path than three other optimization algorithms when solving this path planning problem.

SR-ICA achieved relatively good optimization results in both the optimization applications of benchmark functions and the application in large-scale high-dimensional problems and robot path planning problems. All of these indicate that the optimization ability of the algorithm is improved by the addition of the spiral rising strategy, which also provides a new idea for the improvement of the optimization algorithms in the future.

## Conclusions

This article proposes a new improved intelligent optimization algorithm SR-ICA based on ICA. SR-ICA adopts a spiral rising mode in the position updating process, which increased the search space of the solution group through such particle motions, and improved the precision of the optimization results. SR-ICA had a stronger ability to avoid local optimal solutions, which improved the global search ability of the algorithm.

**Table 12:** Path optimization results.

Algorithm	The path distance
PSO	70
ICA	70
WOA	74
SR-ICA	68

**Table 13:** Parameters settings of the optimization algorithms.

Algorithm	Parameter Settings
ICA	nPop=100; nEmp=5; alpha=1; beta=2; zeta=0.1; MaxIt=150 pRevolution=0.05; mu=0.1
WOA	*N* = 100; MaxIt=150; *e* = 2.717; *b* = 1; *P* = 0.5
PSO	*N* = 100; MaxIt=150 Vmax=5; wMax=0.9; wMin=0.4; c1=2; c2=2
SR-ICA	nPop=100; nEmp=6; alpha=1; zeta=0.1; MaxIt=150 pRevolution=0.05; mu=0.1; *e* = 2.717; *b* = 5; *P* = 0.5

From the experimental results of 19 benchmark functions, it can be seen that SR-ICA had higher search accuracy of optimal solutions, faster search speed and higher stability of search results. In addition, SR-ICA was more suitable for handling high-dimensional and large-scale problems because of its own search mechanism, and the search accuracy did not decrease significantly with the increase of dimensionality, which provides an improved new idea for solving this kind of optimization problems.

The SR-ICA proposed in this paper achieves better optimization results in both mathematical models and engineering optimization problem. In future studies, we will focus on reducing the temporal complexity of SR-ICA. When solving optimization problems, we desire faster solution speed, therefore, we will conduct research aimed at reducing the time-complexity of SR-ICA, and hope that the optimization performance of SR-ICA can be further improved.

##  Supplemental Information

10.7717/peerj-cs.1075/supp-1 Supplemental Information 1 SR-ICA code

10.7717/peerj-cs.1075/supp-2 Supplemental Information 2 ICA code

10.7717/peerj-cs.1075/supp-3 Supplemental Information 3 WOA code

10.7717/peerj-cs.1075/supp-4 Supplemental Information 4 PSO code

10.7717/peerj-cs.1075/supp-5 Supplemental Information 5 DE-ICA code

10.7717/peerj-cs.1075/supp-6 Supplemental Information 6 BSO code

10.7717/peerj-cs.1075/supp-7 Supplemental Information 7 DE code

10.7717/peerj-cs.1075/supp-8 Supplemental Information 8 GWO code

10.7717/peerj-cs.1075/supp-9 Supplemental Information 9 TLBO code

10.7717/peerj-cs.1075/supp-10 Supplemental Information 10 SCA code

10.7717/peerj-cs.1075/supp-11 Supplemental Information 11 Optimization comparison results of intelligent optimization algorithms

## Additional Information and Declarations

### Data Availability

The following information was supplied regarding data availability: The Raw data, all optimization algorithms and programs are available in the Supplemental Files.
